# Social media “SoMe” in neuro-oncology: a review of the literature

**DOI:** 10.1007/s11060-024-04845-6

**Published:** 2024-10-14

**Authors:** Benjamin R. Klein, David J. Levi, Ashish H. Shah, Michael E. Ivan, Allan D. Levi

**Affiliations:** 1 The University of Toledo College of Medicine and Life Sciences, Toledo, OH USA; 2 The University of Miami Miller School of Medicine, Miami, FL USA; 3 Department of Neurological Surgery, University of Miami Miller School of Medicine, Lois Pope Life Center, 1095 NW 14th Terrace (D4-6), Miami, FL 33136 USA; 4 Sylvester Comprehensive Cancer Center, University of Miami Miller School of Medicine, Miami, FL USA

**Keywords:** Social media, SoMe, Neuro-oncology, Neurosurgical oncologist, Neurosurgery, Influencer

## Abstract

**Purpose:**

This article examines the current state of social media (SoMe) in neuro-oncology and neurosurgical oncology. The goal of this paper is to provide thorough discourse regarding benefits and disadvantages of being a neurosurgical oncologist on SoMe, while discussing the place SoMe will have in cranial tumor-based practices going forward.

**Methods:**

The author’s performed a rigorous literature review on the topic. Included information was pertinent to the history of SoMe in neurosurgical oncology and its impact on the field of neuro-oncology. Incorporated as well are the benefits of being a neurosurgical oncologist on SoMe, the drawbacks of participation on SoMe platforms, and knowledge that facilitates discussion about the future of SoMe in neurosurgical oncology.

**Results:**

SoMe plays an important role in neuro-oncology and neurosurgical oncology. SoMe continues to exponentially grow in the healthcare sphere as more providers utilize SoMe platforms. We report objective negative and positive outcomes of SoMe in neurosurgical oncology and neuro-oncology. Here, we summarize these results and provide dialogue describing the effect SoMe is having on the many different aspects of neurosurgical oncology and neuro-oncology.

**Conclusion:**

Although SoMe platforms improve social presence and patient outreach, the use of SoMe can also adversely affect one’s career by exposing clinicians to unchecked societal, legal and professional consequences. While using SoMe as a vessel to propagate career initiatives, neurosurgical oncologists should exercise caution with the content they choose to circulate.

## Introduction

**Fig. 1:**
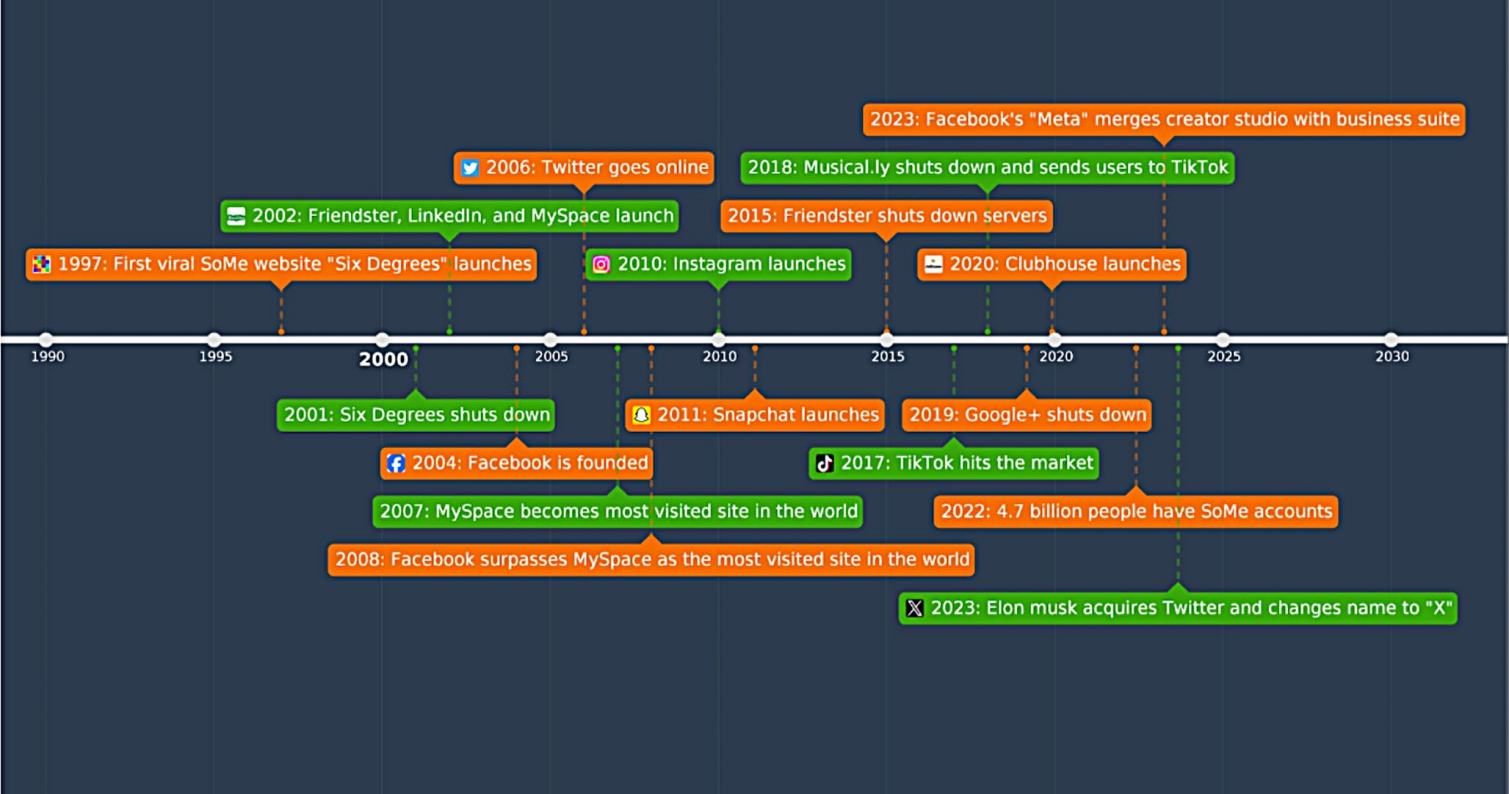
An illustration demonstrating the history of various social media (SoMe) organizations seen in our society. The first SoMe website, “Six Degrees” was founded in 1997. Since then, there have been many different SoMe organizations created [45, 5]

Social media (SoMe) has come to be a part of many people’s daily lives. Merriam-Webster defines SoMe as any type of electronic communication through which users create online communities to share information, ideas, personal messages and other content [1]. The first SoMe website “Six Degrees” was founded in 1997 (Fig. 1.), and since then, there have been many SoMe organizations created [2]. Some common SoMe platforms used today include X, Instagram, Youtube and Facebook. Several of these platforms have millions of active users, with Facebook boasting the most at 3 billion [3]. Worldwide, there are over 4.8 billion SoMe users, with 70% of Americans reporting that they use some variation of a SoMe platform [4, 5]. Usage has also increased quickly, with the percentage of adults using SoMe rising significantly from 2005 (5%) to 2019 (79%) [6]. Interestingly, usage has expanded the most in elderly and ethnically underrepresented individuals [7].

SoMe utilization has continued to be attractive to a wide variety of people because of its potential to influence business, politics and socialization [7]. As one can imagine, healthcare has also been impacted significantly due to the utilization and consumption of SoMe. Currently, more than 40% of healthcare consumers utilize social media for health care information [8]. SoMe has increased interaction amongst clinicians and patients [9]. Concurrently, a study conducted by Hameed et al. found that 70% of physicians surveyed (650) from the top 10 U.S. hospitals per the U.S. News and World Report in 2021 had at least one SoMe profile [10]. SoMe has been utilized by physicians for access to educational resources and references, the recruitment of potential patients for clinical trials, the organization of public health campaigns, and much more [11].

In the field of neurosurgical oncology, the use of SoMe has assumed a larger presence. Ban et al. stated that the SoMe will be a necessity for marketing, patient follow-up, quality improvement, and development in the academic arena [12]. Specifically, neurosurgical oncologists have increasingly utilized SoMe via multiple platforms. Neurosurgical oncologists on SoMe are fond of using certain spaces to grow their professional identity and disseminate research [13]. However, there are pitfalls, such as the reliability of information publicized and patient privacy issues [14]. In this article, we analyze the current state of SoMe in neurosurgical oncology. We furthermore explore how benefits and pitfalls of its use has impacted this specific field, and the field of neurosurgery in totality.

### Search criteria

**Fig. 2:**
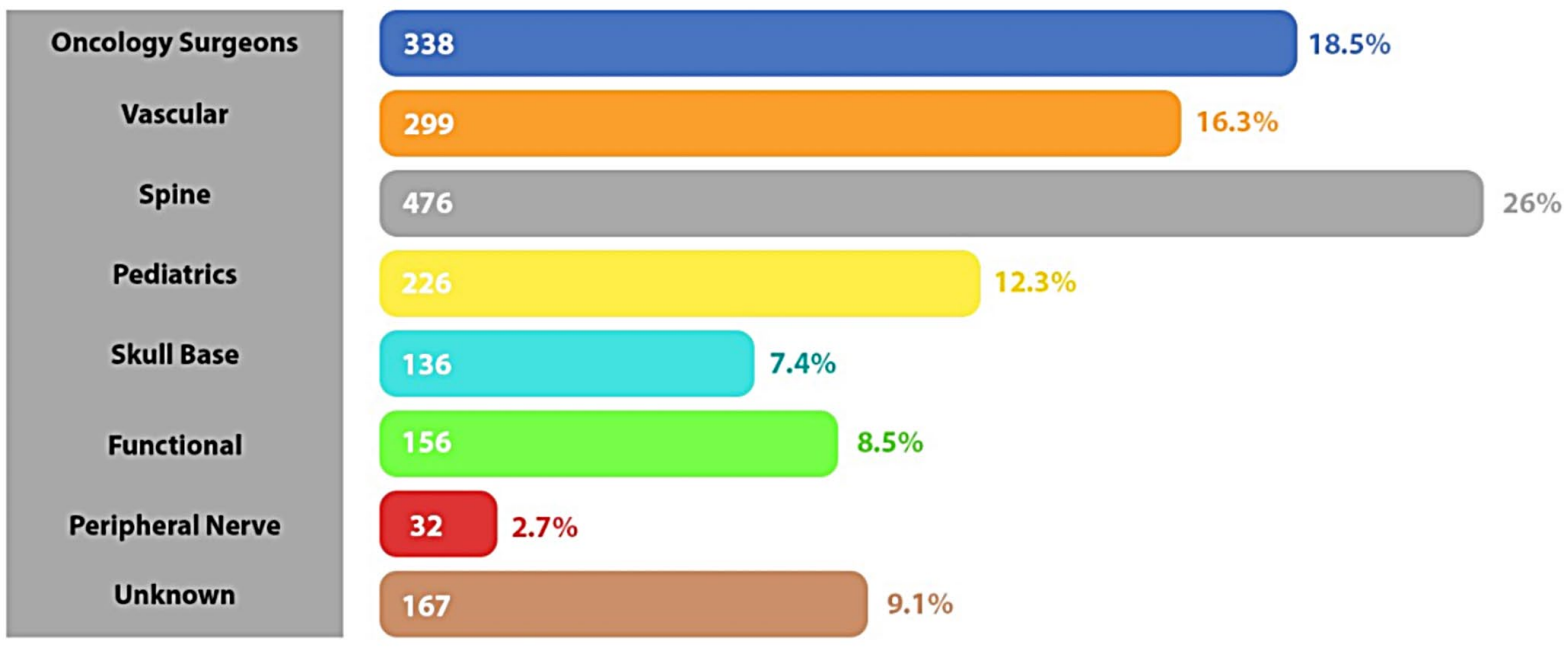
An illustration of data presented by Elarjani et al. showing SoMe representation by subspecialty in neurosurgery. Oncology, spine and vascular have the highest prevalence on SoMe compared to other neurosurgeons [17]

**Fig. 3:**
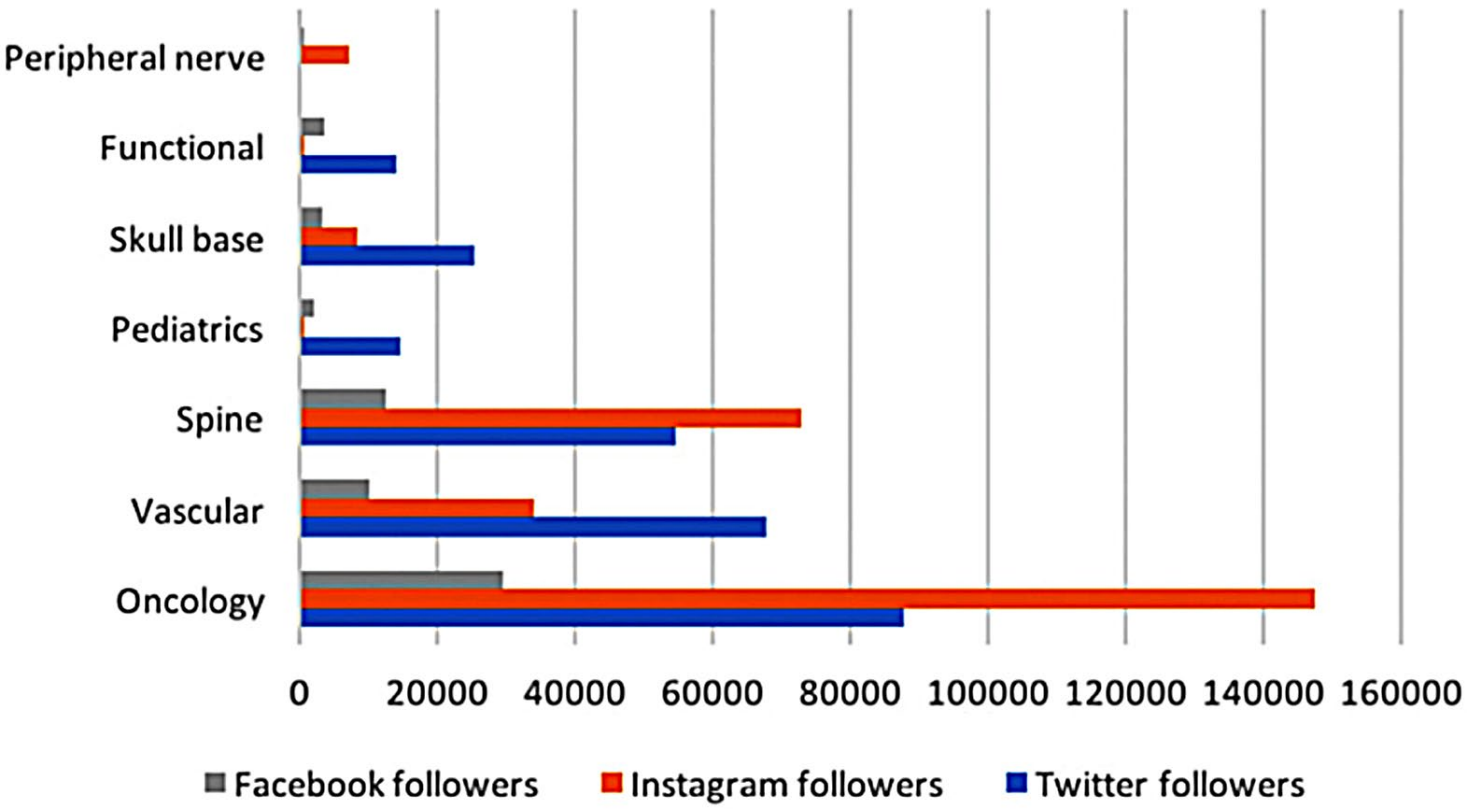
Number of Twitter, Instagram, and Facebook followers in relation to the subspecialty type. Reprinted from World Neurosurgery, Vol. 168, Elarjani et.al,”*Social media presence across U.S. neurosurgical residency programs and subspecialties*”, e43-e49, Copyright (2022), with permission from Elsevier

The articles for this manuscript were obtained via a combination of searches involving, but not limited to, the nomenclature, “neuro-oncology”, “neurosurgical oncologist”, “neurosurgeon”, “social media”, “influencer” “medical influencer”. Inclusion criteria included peer-reviewed manuscripts that gave discourse or data directly relating to the use of SoMe in neuro-oncology or neurosurgical oncology. Consideration was also given to manuscripts that discussed the state of SoMe in medical providers overall, but included sources had to provide value to the discussion as it related to SoMe in neuro-oncology or neurosurgical oncology. Exclusion criteria included those manuscripts that did not emphasize the state of SoMe in neuro-oncology or neurosurgical oncology, as well as those that held a brief discussion about SoMe in either of these fields but lacked adequate evaluation of its impact. If an article met these standards and was deemed relevant to the context of our manuscript, it was included. Each author followed these guidelines and used the academic search engines PubMed and Google Scholar to assemble manuscripts relevant to our topic. In total, 70 manuscripts were obtained between Oct. 2023 to Apr. 2024. In congruence, the authors agreed that the 44 represented references in this paper be included in our discussion. Figure 1 in this manuscript was an adaptation from the cited source, while Figs. 2 and 3. have been adapted with permission from the appropriate journal. Given heterogeneity of literature included, the authors did not use a specific system to assess the risk of bias of articles used, however, the nature of data being discussed is primarily objective metrics represented in manuscripts that were peer-reviewed. In addition, multiple authors independently reviewed included articles in a iterative process to reduce the risk of selection bias.

### Neurosurgical oncologists on social media

The development of SoMe throughout recent years has allowed broad access to the academic arena [15]. Specifically, it has allowed insight into previously more private professions such as neurosurgery [15]. For example, the Journal of Neurosurgery’s Instagram page, which has 48,000 followers, commonly shares excerpts from recently released manuscripts [16]. Hameed et al. showed the social media presence of neurosurgeons by platform from an individual perspective. They found that amongst surgeons, neurosurgeons compared similarly against other surgical specialties in terms of their presence on social media [10]. Neurosurgery was represented on all of the SoMe sites being surveyed, with the SoMe platform represented most being LinkedIn (52%), followed by X (formerly Twitter) and Facebook (each with 18%) [10].

Specifically, neurosurgical oncology is a subspecialty in neurosurgery that is more active on SoMe. Elarjani et al. surveyed 110 academic programs and 1900 faculty and discovered that neuro-oncology and neurovascular specialists have the largest presence on SoMe compared to the other subspecialities of neurosurgery (Fig. 2. and 3.) [17]. Furthermore, the authors showed that neurosurgical oncologists and neurovascular neurosurgeons were more likely to have Twitter accounts, whereas neurovascular and skull-base neurosurgeons are more likely to have an Instagram account [17]. Lastly, neurovascular surgeons had the most tweets, followed by neurosurgical oncologists and spine surgeons [17]. Overall, Haider et al. found that neurosurgical oncologists under 50 are more active on SoMe [13]. They also showed that benefits of SoMe use as a neurosurgical oncologist, such as an increased number of patient referrals, was positively correlated with the number of followers a surgeon has [13].

### Benefits of being a neurosurgical oncologist on social media

There are many reasons why neurosurgical oncologists adopt SoMe platforms. Neurosurgery is a discipline practiced all over the world, and a pertinent reason why neurosurgeons in general are joining SoMe platforms is to get a realistic take on how neurosurgery is performed in different economic and cultural backgrounds [18]. For example, treatment options, new surgical equipment, end-of-life care, and use of resources can be easily shared and discussed [18]. SoMe allows a perspective of global neurosurgery that was not as easily accessible before, free from conventional filters represented in certain top-down modalities [18]. In neuro-oncology and neurosurgical oncology, some choose to use SoMe because they find it easier to consult patients and collaborate, increase awareness of certain public health campaigns, and share new study findings.

A study by Alvi et al. explored SoMe impact on glioblastoma education and healthcare providers ability to interact with large audiences regarding this topic. Researchers of this study analyzed over 1600 X posts from a 1000 different accounts and found the majority of users talking about glioblastoma were considered a “MD” or “researcher” at 54% [19]. Most of these X posts featured links to novel patient resources, scientific discoveries, and newly published research articles that provided new information for patients to consider regarding glioblastoma [19]. Patients find this information helpful, and also find comfort in interacting with other SoMe community members experiencing tumor diagnosis either directly or indirectly [19]. Leaders in neurosurgical oncology realize the value of using SoMe to interact with a large patient audience in this way as well. This is reflected by the increase in followers institutions have seen on SoMe, such as The Congress of Neurological surgeons (CNS) Tumor Section, which launched its X account in 2017 and has since accumulated over 5000 followers [19]. A reason why patients and neurosurgical oncologists alike seem to enjoy this delivery method for information pertaining to tumors is because of low character limits and visual aid that can be provided on many SoMe platforms, which lend to easily understandable medical information that is useful to patients [19, 20].

Promotion of neuro-oncological campaigns are widely distributable with SoMe, which is another benefit. National Brain Tumor Society (NBTS) advertises the “Go Gray in May” campaign nationally to bring awareness, support, and empower those impacted by brain tumors. The group fundraises for research purposes, holds events for the community to connect, and provides opportunities for individuals to learn more about tumor related issues. SoMe also presents new opportunities for researchers in neuro-oncology and neurosurgical oncology to connect with one another, while also allowing the opportunity to connect with clinicians, politicians, the public, and other stakeholders on a global level [21]. Efforts have been started along this line to increase collaboration between neuro-oncologists through NCI-CONNECT, a program created by Dr. Mark Gilbert MD and Dr. Terry Armstrong PhD at the National Cancer Institute. This program has the goal of increasing understanding about central nervous system tumors through connecting providers in the field of neuro-oncology, so that they may share novel research, treatment modalities and case presentations [22]. Furthermore, NCI-CONNECT utilizes SoMe to reach a large patient population, so that they are aware of the newly discovered information [22]. Similarly, the “#BTSM Chat” account on X has over 2900 followers and is active in producing content directed at educating users about different topics relating to tumors.

Research output by neurosurgeons on SoMe has been reflected by the increased academic metrics in both neurosurgical departments and journals. Riccio et al. showed Pearson Correlation coefficient of 0.35 with a p value of 0.0003 between a neurosurgeon’s twitter “influence” rank and h-index score [23]. Similarly, Cloney et al. found that neurosurgery departments that were identified as “influencers” (per their protocol) had more departmental and institutional NIH funding, as well better Doximity rankings and better affiliated medical school ranking [24]. They also reported that the number of X followers specifically had the strongest correlation with academic metrics [24]. Simultaneously, Alotaibi et al. revealed that the 11 neurosurgical journals out of the 38 they analyzed with a SoMe presence had greater academic metrics compared to the ones that did not [25]. The average H-index score for those 11 journals was 86 while an independent analysis of 53 journals with a neurosurgical focus found an average h-index of 54 [25, 26]. Riccio et al. demonstrated a similar finding in their study analyzing the top 100 neurosurgeons on Twitter. They found that the average h-index was 27.6 ± 19.7, with a median of 24 (range 1-104) [23]. The p-value of this data was 0.0003, with a pearson correlation coefficient of 0.35 [23].

### Potential negative outcomes from using SoMe as a neurosurgical oncologist

**Fig. 4:**
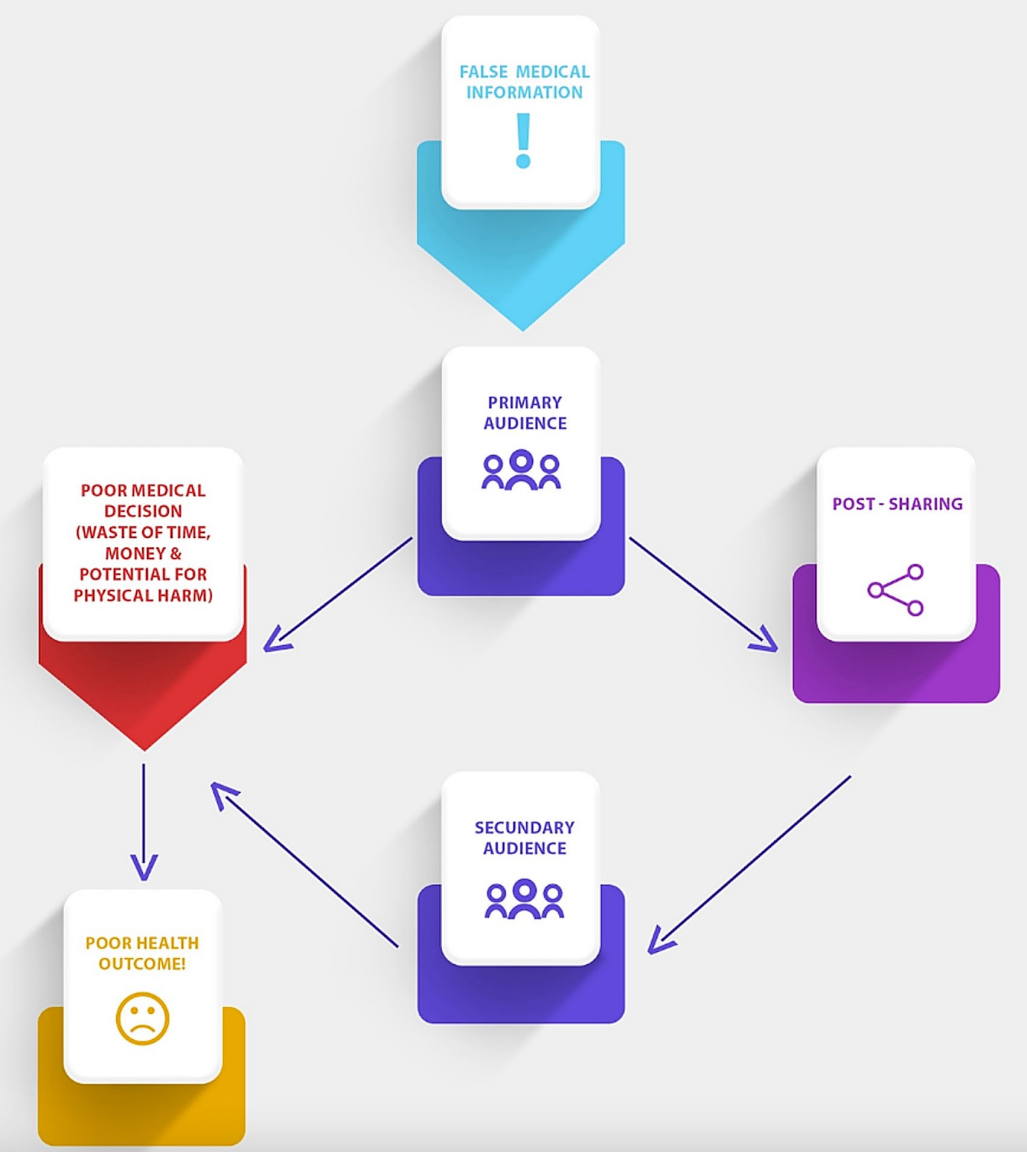
A visual representation showing how false medical information can lead to poor health outcomes. This information can not only negatively impact the primary audience that sees the information originally but can also impact a secondary audience that sees the information through “sharing methods” made capable by SoMe platforms. False medical information on SoMe has the capability to disseminate and impact populations at large

An area neurosurgical oncologists’ and those in neuro-oncology should be cautious of when using SoMe is the circulation of false information regarding case information, patient outcomes, and treatments. Unlike peer-reviewed journals that subject articles to rigorous scrutiny and peer review before acceptance for publication, SoMe only requires that you have an audience to distribute information [27]. One study examined 15,852 Facebook posts relating to cancer information and found that 19% of those posts contained scientifically inaccurate information regarding cancer etiologies and treatment [28]. Another example is the dissemination of alternative oncology, which promotes the use of false medications, miraculous diets, electronic diets, and psychic therapies that lead to unrealistic expectations about cancer treatment [29]. Similarly, in 2016, 20 scientific articles were released that included false or unsubstantiated cancer cures and claims that a cancer cure exists but the government sequesters them for financial benefit [30]. Sadly, 37% of Americans surveyed on their beliefs of these claims maintained that they believe the FDA is organizing efforts to hide a cure to cancer [30]. These examples, and many others including false adverse effects of immunization etc., are not only misleading to an influenced audience, but could also potentially lead to physical harm in that same patient population. Quality of research is concern for some as well, with 62% of neurosurgeons reporting that they would not be involved in any project or paper that included the use of SoMe to collect data [31]. We illustrate this idea in Fig. 4. False medical information can reach a primary audience through SoMe, that can lead to poor medical decisions and thereafter poor medical outcomes (Fig. 4.). Furthermore, the capability of a primary audience to share that false medical information with their peers (i.e. a secondary audience) compounds this negative potential (Fig. 4.). To avoid spreading misinformation, neurosurgical oncologists should be diligent to include content that propagates succinct and comprehensible information such as the pathophysiology of disease, relevant treatment options, risk of operative intervention, and other pertinent knowledge to the field of neurosurgery.

Another risk of using SoMe is the potential to post content that is deemed unprofessional. A study surveyed 600 medical school admissions officers in the United States and found that 9% would regularly check applicants’ SoMe presence, and half said that the professionalism of their content was a contributing factor to the decision of acceptance [32]. Also, a study found that 12.2% of 390 residents analyzed had clearly unprofessional content (binge drinking, HIPAA violations, etc.) visible on their Facebook page [27]. Disappointingly, the same authors found in a follow-up study in 2015 that 5.1% of attending surgeons evaluated had similar content visible on their SoMe pages [27]. Similarly, a recent article by Alan et al. analyzing spine surgeons’ use of SoMe highlighted these issues and emphasized that a potential downfall that neurosurgeons may fall into is distributing information that is more so for entertainment instead of education [33]. They also explain that spine surgeons can encroach on professionalism by exploiting treatment outcomes and displaying a life that is not a realistic one they live [33]. Respecting patient privacy and confidentiality is essential in fostering a positive physician-patient relationship and garnishing trust from the community in medical professionals [34]. However, privacy and censorship are at risk via the use of SoMe. Health care workers are well informed about laws regarding patient confidentiality, however, 40% of health care workers were unaware of their workplace policy regarding SoMe use according to a study by Surani et al. It is easy for SoMe use to transcend professional boundaries and lead to unprofessional behavior, an irreversibly infamous online image, and potentially legal consequences [35]. A prominent example of compromised patient information through SoMe is with the #ShareAStoryInOneTweet movement in May of 2018. The movement was kickstarted by a physician who shared a story of one of their patients and many followed suit, leading to posts that contained sensitive information that put certain physicians’ careers at risk while also risking harm to patients [10]. One post in this movement that contained personal identifiable information gained 13,491 retweets and 55,994 likes before being deleted, highlighting the efficiency and danger of discussing patient cases on SoMe platforms [10]. In recent years, mass social media movements such as this one are making more frequent appearances and perpetuate the risk of violating the doctor-patient relationship through the misuse of SoMe platforms [34]. These reasons, as well as the other negative risks mentioned, should make neurosurgical oncologists cautious when using SoMe so that they do not be portrayed as untrustworthy or unprofessional.

## Discussion

Although the use of SoMe for disseminating medical information is in its infancy, more than 40% of healthcare consumers utilize social media for health care information [8]. As with other specialties, neuro-oncologists and neurosurgical oncologists will be impacted by the continued growth of SoMe into the healthcare sphere. The growing percentage of the population using social media, adoption of SoMe use by major medical institutions, and the younger average age of physicians using SoMe is valuable evidence of the impact SoMe is already having in healthcare [8]. For example, the Department of Veteran Affairs VistA healthcare system allowed its physicians to communicate online with its over 3 million patients and found that the implementation has cut healthcare costs by 30% due to patients being more engaged and care being provided in a timely manner, along with avoiding unnecessary testing and procedures [36]. Claus et al. demonstrated the effectiveness in reaching a large patient population through SoMe in the field of neuro-oncology by using certain platforms to recruit patients for their Yale acoustic neuroma (AN) study. The researchers of this study state they enrolled 1024 participants in their AN study and the majority of those enrolled heard about the study through various patient organizations (ABTA, NBTS, etc.) SoMe platforms [37]. Of note, they noted how the use of SoMe in the recruitment of these patients greatly reduced overhead costs of their study [37]. Neuro-oncologists and neurosurgical oncologists have a great opportunity through various SoMe platforms to grow their practice and increase their presence nationally and globally in a cost-effective way.

Another perspective to note is that there have been many SoMe platforms and apps that have gone offline suddenly citing potential issues with website stability and sustainability. A major example highlighting this potential issue is the downfall of the prominent SoMe app “Vine”. Vine (2012–2017) was a popular SoMe app used by a broad audience that included short videos of a few seconds, with content covering everything from comedy to education. Jessica Maddox, assistant professor at the University of Alabama College of Communication and Information Sciences, explained in an article for the MIT Technological Review that although there were a variety of reasons why Vine was shut down, chief amongst them was the lack of monetization and ad possibilities [38]. These issues are becoming increasingly prevalent for other SoMe platforms, with “Content Creator” becoming a legitimate career, it is impossible to have a SoMe platform today to exist without monetization opportunities for prominent users and this potentially contributes to instability of certain apps [38]. Changes such as these have a massive impact on the audience, the type of content being distributed, and compensation. Although there are many perks to being on SoMe, a neuro-oncologist or neurosurgical oncologist with a large platform should be aware of this possibility. If a provider begins to rely too much on a singular SoMe platform, there is a chance that they may lose that avenue providing patient recruitment, advertising, and even possibly compensation if that SoMe website were to go offline. Neuro-oncologists and neurosurgical oncologists should be diligent to try and build platforms on multiple, structurally sound platforms with proven durability if they wish to enhance their practice and image through this method.

Important to consider as well, specifically in the field of neuro-oncology and neurosurgical oncology, is how SoMe provides a place for patients, loved ones, and friends experiencing the consequences of tumor to connect with a community where they can find new information and comfort. Tripathi et al. analyzed how individuals used the prominent SoMe platform Reddit to get information regarding different neurologic lesions. They found that of the top 100 posts relating to brain tumors, most were about glioblastoma and were individuals writing about their loved ones’ experiences [39]. The top three themes of content that emerged were harnessing of hope, appreciation of support from the SoMe community, and guidance on moving through the grief process [39]. They observed that most posts ended with offering a “listening ear” for others in the community, displaying the support and understanding the individuals in these groups have for each other [39]. A quote from an individual post in this paper states, “*This subreddit*, *silly as it sounds*, *has been a God send... Even if no one reads this I want to thank you all for sharing here. I was alone and stunned sitting in my garage and not knowing what to do and you all gave me such insight and hope* [39]. Clinically, it may be important for neurosurgical oncologists to offer SoMe platforms as a potential outlet for patients and their families to connect with others going through similar experiences. Additionally, a neuro-oncologist or neurosurgical oncologist with a platform might offer patient experiences and outcomes on their own platforms as a safe-space for patients and loved ones to interact and so that they may be able to interact with them.

Future implications of SoMe in the medical field are becoming increasingly important to consider as platforms continue to evolve and expand. One being the prevalence of mass disease, and the emotional influence SoMe can have on patient populations at large. Specifically in neuro-oncology, Mrugala et al. reported that those in the field were severely impacted in many ways due to the COVID-19 pandemic. They state that 94% of 582 respondents in a survey they conducted reported changes in their clinical practice [40]. 10% reported that they still felt the need to see patients in person due to billing concerns and pressure from their institutions [40]. Furthermore, 68% reported suspending enrollment for at least one clinical trial, with 62% stating they suspended phase III enrollment [40]. It is interesting to ponder how organized SoMe efforts could have impacted these issues and more that neuro-oncologists faced in the early stages of the pandemic. Limitations such as the amount of credible material published on this topic and SoMe being relatively new in the healthcare setting are acknowledged by the authors. The authors also acknowledge the inherent selection bias possible from not performing a systematic review of the literature regarding the subject matter, however, the nature of data being discussed includes only objective metrics from peer-reviewed articles and minimizes the risk of bias by inclusion and exclusion criteria. Overall, however, SoMe being used on a regular basis in the healthcare setting is becoming the norm and physicians, institutions, and hospitals alike should take advantage, but should proceed with caution when utilizing its resources for the betterment of a career or the well-being of patients.

## Conclusion

Since its inception, SoMe use has exponentially grown over the last 20 years [2, 6]. The healthcare field is no exception. Neurosurgical oncologists, and neuro-oncologists in general, have been increasing their presence on SoMe [22, 28, 29, 37, 39, 41]. There are many beneficial reasons as to why neuro-oncologists and neurosurgical oncologists would want to join SoMe, including the broad dissemination of research, exchange of clinical knowledge, ability to educate and connect with patients, and more [16, 18, 42, 43]. However, there are drawbacks of SoMe that should lead to caution, such as the potential to be viewed as untrustworthy and unprofessional, all while adopting the capability to be publicly scrutinized and waste valuable time on various different platforms [14, 27, 31, 32, 35, 44].As SoMe continues to grow and expand into the many different areas of healthcare, neurosurgical oncologists and neuro-oncologists alike posting on SoMe platforms should remain diligent in making sure that their content is professional, informational, and beneficial to their potential patients, as well as to their neurosurgical colleagues and all those interested in the field throughout the world.

## Data Availability

Data was analyzed from external sources for discussion. Instructions on how to access these sources in in the manuscript. This review was not registered and a review protocol was not prepared. All material in this manuscript is available via reference search on PubMed or Google Scholar.
